# Swallowing Function Evaluation in a Patient with Gerstmann-Sträussler-Scheinker Disease with Pro105Leu: A Case Report

**DOI:** 10.3390/ijerph18189734

**Published:** 2021-09-15

**Authors:** Ayako Nakane, Shohei Hasegawa, Miki Ishii, Tomoe Tamai, Rieko Moritoyo, Mitsuko Saito, Mariko Ando, Haruka Tohara

**Affiliations:** Department of Dysphagia Rehabilitation, Graduate School of Medical and Dental Sciences, Tokyo Medical and Dental University, 1-5-45 Yushima, Bunkyo-ku, Tokyo 113-8549, Japan; shoheihasegawa.dent@gmail.com (S.H.); mickey.cookie05015@gmail.com (M.I.); yagadawooooo@gmail.com (T.T.); r.moritoyo.swal@tmd.ac.jp (R.M.); mitsuko_0331@msn.com (M.S.); 0401eclat@gmail.com (M.A.); h.tohara.swal@tmd.ac.jp (H.T.)

**Keywords:** Gerstmann-Sträussler-Scheinker disease, swallowing function, oral ingestion

## Abstract

Gerstmann-Sträussler-Scheinker disease (GSS) is a genetic prion disease. Swallowing function evaluation in patients with GSS remains unclear. Here, we describe a case of videofluoroscopic examination of swallowing (VF) to facilitate continued oral ingestion in a patient with P105L GSS. A 67-year-old woman developed gait disturbance and Parkinsonism symptoms at the age of 54 years. Since her family wanted her to continue oral ingestion, we performed VF, which revealed impairment and preservation of the oral and pharyngeal phases, respectively. Moreover, the impairment of the oral phase was improved by adjusting the patient’s posture and food consistency. A swallowing function evaluation based on the condition of a patient with GSS may facilitate continued oral ingestion.

## 1. Introduction

“Prion diseases” is the general term for diseases caused by abnormal prion proteins and currently lacks an effective treatment method. The annual incidence rate of human prion diseases is approximately 1–2 people per million; moreover, they are classified into idiopathic, genetic, and acquired prion diseases depending on the origin of the responsible prions. Gerstmann-Sträussler-Scheinker disease (GSS) is a genetic prion disease. GSS with a P105L mutation is rare, with only 10 reported cases in Japan and the UK [1,2]. P105L GSS is characteristically observed in Japanese individuals; patients present with symptoms including characteristic Parkinsonism and spastic paraplegia, exhibiting gradual progression of gait disturbance and dementia within approximately 5–10 years, until the patient is in a state of akinetic mutism by the end stage [3].

There have been reports regarding patients with P105L GSS whose swallowing function has deteriorated until enteral nutrition or gastrostomy is required [2,4]. However, there have been no reports regarding detailed swallowing evaluations before this state has been reached. Furthermore, the pathophysiology of the associated dysphagia remains unclear. Gastrostomy is seldom chosen for patients with prion diseases due to infection control; instead, a nasogastric tube is used in most cases. Here, we report the use of videofluoroscopic examination of swallowing (VF) [5] to allow for continued oral ingestion in a patient with P105L GSS.

## 2. Case Report

A 67-year-old female nursing home resident (Tokyo, Japan) developed gait disturbance and Parkinsonism symptoms at the age of 54 years. After 3 years, her Parkinsonism symptoms worsened; moreover, she presented with declined cognitive function. Genetic testing yielded a diagnosis of P105L GSS. Furthermore, she had four sisters with the same disease. At 8 post-onset years, the patient was using a wheelchair and had communication difficulties. She continued oral ingestion; however, she was admitted to an institution. By 12 post-onset years, she could not fully open her mouth or eat solid food; accordingly, she was placed on a paste diet. At 13 post-onset years, she presented with apparent tooth clenching and bruxism became apparent. Moreover, she had to be fed in a reclined position using a syringe and took over 30 s to swallow 2 mL. She lost 5 kg of body weight within 2 months. However, since her family wanted her to continue oral ingestion, the patient underwent VF, a gold standard test for assessing aspiration.

A clinical examination revealed that the patient had a Barthel Index of 0 points (basic activities of daily living requires full assistance), Functional Assessment Staging at stage 7, Mini-Mental State Examination with 0 points (severe cognitive impairment), and Food Intake LEVEL Scale level of 7 (impossible to talk and move) [6], with apparent cervical rigidity. Although she occasionally opened her eyes, she could not communicate. She made slight mouth-opening movements during feeding. Even after placing food in the oral cavity, her tongue only moved in the anterior–posterior direction, which indicated that part of the bolus was pushed from the mouth, with only a small amount being transported into the pharynx.

VF was performed using 5 mL of an extremely thick paste in the seated position, and 5 mL of a moderately thick paste as well as 5 mL and 7 mL of an extremely thick paste when reclining at 30°.

**Figure 1:**
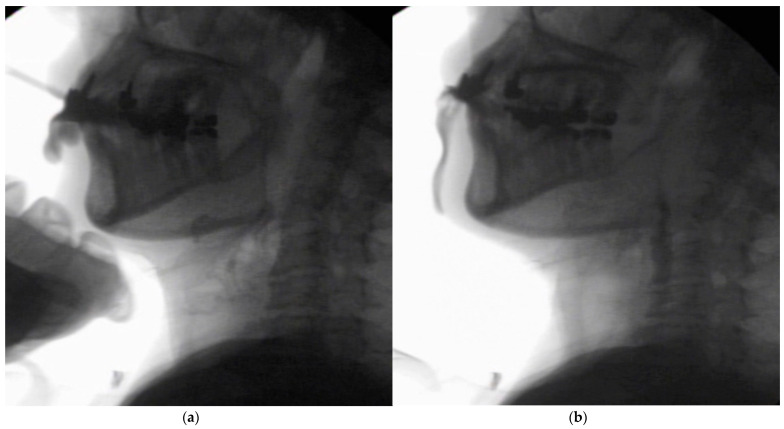
(**a**) Videofluoroscopic examination of swallowing (VF) with the patient sitting up. The test food was 5 mL of an extremely thick paste. Most of the food dribbled out of her mouth, with only a small amount being transported to the pharynx. (**b**) Maximum hyoid elevation upon deglutition.

**Figure 2:**
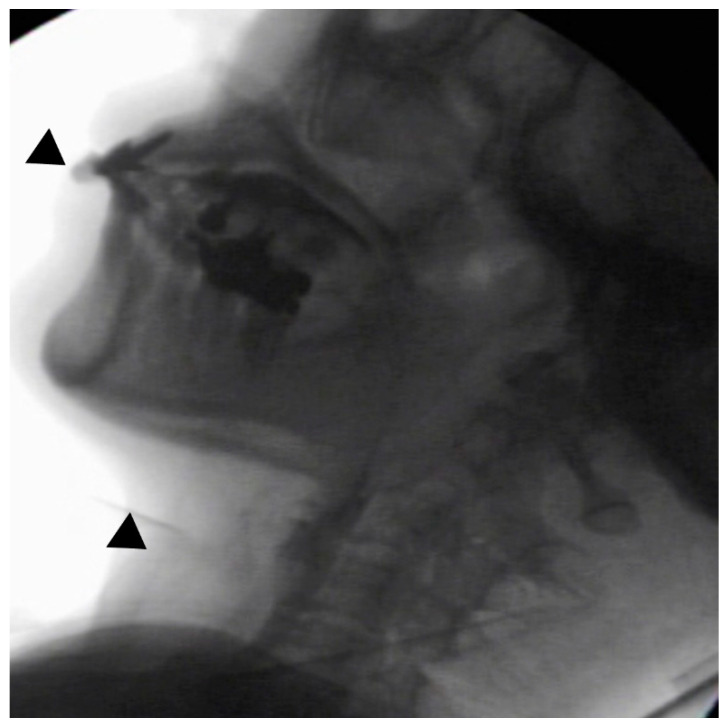
The patient was reclined at 30° for the administration of 5 mL of an extremely thick paste. Oral transport was better than in the sitting position; furthermore, a little of the food dribbled out of the mouth (above ▲), and no aspiration occurred (below ▲).

**Figure 3:**
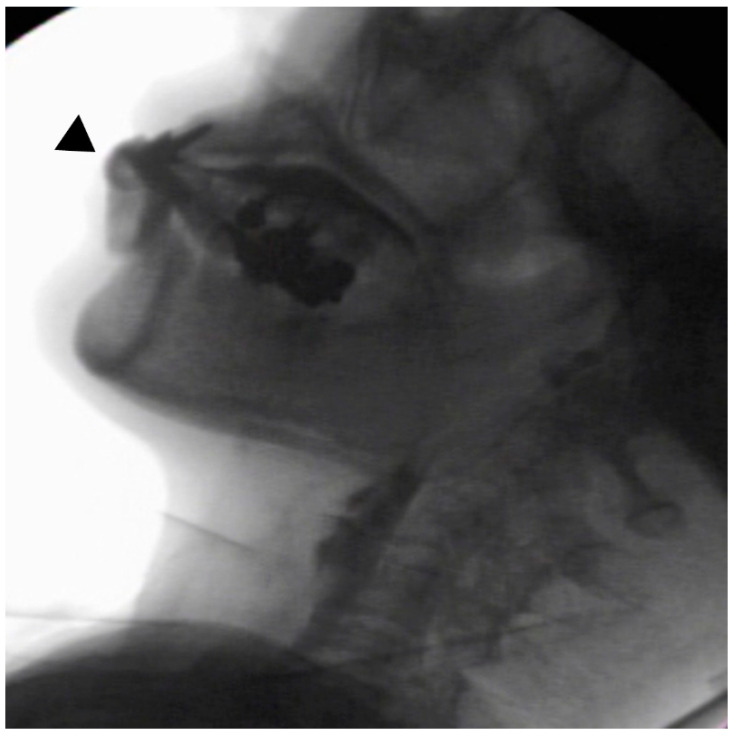
The patient was reclined at 30° for the administration of 7 mL of an extremely thick paste. Even when the food was placed in the retromolar area, most of the food dribbled out of her mouth (▲) upon triggering her swallowing reflex.

**Figure 4:**
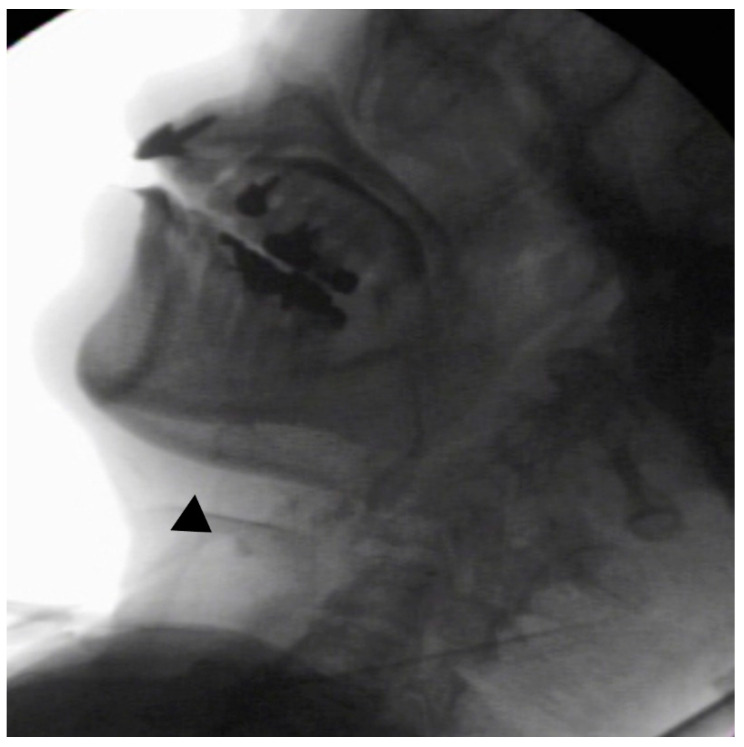
The patient was reclined at 30° for the administration of 5 mL of a moderately thick paste. There was delayed triggering of the swallowing reflex; moreover, silent aspiration occurred (▲).

In the seated position, spoon-feeding was infeasible and bolus transport did not occur; furthermore, anterior–posterior tongue movement caused most of the food to dribble out of her mouth. After placing food on top of her tongue using a tube-fitted syringe, most still dribbled out of her mouth (Figure 1a); however, a proportion was transported into the pharynx (Figure 1b). Furthermore, after placing food at the back of her tongue with the patient reclined at 30°, a small amount still dribbled out of her mouth; however, the patient could swallow without aspiration (Figure 2). After increasing the amount placed in her mouth at one time to 7 mL, more food dribbled out of her mouth (Figure 3); additionally, there was a decreased amount transported to the pharynx. When 5 mL of a moderately thick paste was placed in her mouth, none of it dribbled out; however, slight silent aspiration was present (Figure 4). There were no particular issues observed due to peristalsis from the upper esophagus to the stomach.

VF time measurements were obtained based on the following criteria. Oral transit time (OTT) is defined as the time required for a bolus to pass across the base of the tongue once swallowing is initiated [7]. Pharyngeal delay time (PDT) begins when the head of the bolus reaches the point where the lower mandible edge crosses the tongue base and ends when laryngeal elevation begins for swallowing completion. Pharyngeal transit time (PTT) is defined as the time elapsed from the point when the head of the bolus reaches the point where the lower mandible edge crosses the tongue base to the moment when the tail of the bolus passes through the cricopharyngeal region or the pharyngoesophageal segment. [8] Pharyngeal clearance (PC) is defined as the bolus head at the fauces to offset the upper esophageal segment (UES) opening. The UES opening time (UOD) is defined as the time between onset and offset of the UES opening. The duration of hyoid movement (DHM) is defined as the time between onset and end of the hyoid movement. Oropharyngeal transit (OT) is defined by the tongue tip being at the incisors to offset the UES opening. Stage transition duration (STD) is defined as the bolus head at the fauces to the onset of hyoid movement (DHM) [9] (Table 1 and Table 2). Our patient demonstrated a prolonged OTT; however, her pharyngeal-stage parameters, including the UOD and DHM, were not inferior to those of healthy adults (Table 1). Additionally, oral stage impairment with preservation of the pharyngeal stage has been described in a patient with prion disease (Table 2).

These results indicated that, to maintain oral feeding, the patient needed to be reclined at 30° with around 5 mL of an extremely thick paste being placed at the back of her tongue for each mouthful.

## 3. Discussion

To our knowledge, regarding swallowing function evaluation in patients with prion disease, there have been only case reports of one patient each with V1801 genetic Creutzfeldt–Jakob–disease (CJD) [12] and MM2-cortical-type sporadic CJD [13]. Moreover, there are no related reports regarding patients with GSS in the Japanese population. Regarding dysphagia in patients with GSS, there have been reports of enteral feeding in a patient with GSS with a mutation in codon 102 who showed severe dysphagia following aspiration pneumonia [14], of gastrostomy in European patients with P105L GSS [4], of enteral feeding in a patient with P105L GSS who showed pseudobulbar palsy [15], of incapability of oral ingestion in a patient who died from aspiration pneumonia [16], and of enteral feeding in another patient who died from aspiration pneumonia [17]. However, these previous studies did not describe the swallowing function. Our case report provides a detailed description of the swallowing function in a patient with P105L GSS at 156 months after onset.

Swallowing function is divided into five stages: anticipatory, preparatory, oral, pharyngeal, and esophageal [18], which involve cognitive function, mastication, bolus transport into the pharynx, the swallowing reflex, and peristaltic movements to the stomach, respectively. Moreover, VF can identify the stages causing problems. In our patient, there were problems in the anticipatory, preparatory, and oral stages since she presented with diminished food recognition, no masticatory movements, and decreased oral transport capacity. Moreover, oral stage impairment with preservation of the pharyngeal stage has been described in a patient with V1801 gCJD [12] and MM2C-type sCJD [13]. None of the aforementioned patients presented with brainstem atrophy. Moreover, a study involving 10 patients with GSS with a missense mutation at codon 105 reported that 4 out of 5 patients who underwent computed tomography or magnetic resonance imaging (MRI) did not present with brainstem or cerebellar atrophy upon respective neuroimaging studies [2]. Taken together, these findings suggest that patients with GSS without brainstem atrophy may not exhibit pharyngeal stage impairment.

We used time measurements of the swallowing dynamics to objectively evaluate our patient’s swallowing function. Furthermore, we compared the measurements obtained with those of healthy adults and older people, with consideration of the textures and volumes of test foods (Table 1 and Table 2). Our patient showed a prolonged OTT; however, pharyngeal-stage parameters, including the UOD and DHM, were not inferior to those of healthy adults. There were neither pharyngeal residues nor aspiration; however, there was severe impairment of the anticipatory and oral stages. Changing the patient’s posture improved the oral stage impairment. The transport time from the tongue tip at the incisors to the bolus head at the fauces was shorter for the moderately thick paste than the extremely thick test paste [19]. However, there was delayed triggering of the swallowing reflex and pre-swallowing aspiration. Aspiration was eventually avoided by changing the test food to an extremely thick paste. Changing the amount per mouthful and its positioning in the mouth also influenced the amount of food that dribbled out of her mouth. When the patient was seated, bolus transport was poor and OT was stretched; thus, it would be worth attempting reclination. If the patient exhibits a delayed swallowing reflex or aspiration of the pharynx, it is worth testing a switch from liquid foods to a concentrated paste (Table 2). This suggests that addressing oral stage impairment may allow for prolonged oral intake.

Safely continuing oral ingestion not only avoids aspiration pneumonia but also may help preserve the bonds between patients who become mute and akinetic at an early stage and their caregivers. Therefore, there is a need to evaluate swallowing function and to provide appropriate guidance.

This study has several limitations. Since the patient is still alive, we did not conduct a pathological analysis. Furthermore, this was a single-patient case report. Finally, we did not conduct an MRI evaluation.

## 4. Conclusions

This case report describes the evaluation of swallowing function in a patient with P105L GSS, which revealed impaired and preserved oral and pharyngeal stages, respectively. Appropriate evaluations of swallowing function and measures to address oral stage impairment may facilitate the continuation of oral ingestion for longer periods.

## Figures and Tables

**Table 1:** Comparison of swallowing function between our patient and healthy adults.

Case	Author	Participants	Age	Sex	Test Food	Volumes (mL)	OTT(s)	PDT(s)	PTT(s)	PC(s)	UOD(s)	OT(s)	STD(s)	DHM(s)
1		Current patient	67	female	paste	5	20.25	0.1	0.43	6.91	0.27	20.42	6.67	0.47
2	Wakasugi [7]	Healthy elderly	70.3 ± 7.8	13 males, 7 females	liquids	10	0.89 ± 0.46	N.D.	N.D.	N.D.	N.D.	N.D.	N.D.	N.D.
3	Santos [9]	Healthy volunteer	55.2 (35–69)	8 males, 7 females	paste	5	0.72 ± 0.73	N.D.	0.38 ± 0.08	0.64 ± 0.22	0.35 ± 0.09	0.94 ± 0.49	0.07 ± 0.21	0.69 ± 0.26
4	Cassani [10]	Healthy volunteer	58 (29–77)	18 males, 12 females	paste	5	0.41 ± 0.28	N.D.	0.23 ± 0.07	0.48 ± 0.27	N.D.	N.D.	N.D.	N.D.
5	Dantas [11]	Volunteers	61 (33–77)	8 males	paste	5	0.39 ± 0.28	N.D.	0.22 ± 0.07	0.42 ± 0.25	0.2 ± 0.04	0.64 ± 0.35	N.D.	N.D.
			53 (29–72)	7 females	paste	5	0.59 ± 0.42	N.D.	0.25 ± 0.07	0.56 ± 0.27	0.22 ± 0.07	0.94 ± 0.58	N.D.	N.D.
6	Yoshiakawa [8]	Dantate elderly	81.2 (80–87)	12 males, 7 females	liquids	10	1.05 ± 0.31	0.16 ± 0.14	0.7 ± 0.15	N.D.	N.D.	N.D.	N.D.	N.D.

OTT: oral transit time; PDT: pharyngeal delay time; PTT: pharyngeal transit time; PC: pharyngeal clearance; UOD: upper esophageal segment opening time; OT: oropharyngeal transit; STD: stage transition duration; DHM: duration of hyoid movement; N.D.: not described.

**Table 2:** The results of swallowing function assessments of patients with prion disease.

Case	Author	Participants	Onset of Age	Sex	Duration of Onset	FILS Level	Nutrition Intake	Meal	Position	Test Food	Bolus Transport	PC(s)	OT(s)	STD(s)	Pharyngeal Swallow	Aspiration
1	Current patient	GSS p105L	54	F	156 M	7	oral	assistance	sitting	5 mL of extremely thick paste	poor	6.91	20.42	6.67	delayed	non
								assistance	reclining	〃	poor	2.47	5.67	2.3	not delay	non
								assistance	reclining	5 mL of moderately thick paste	poor	7.68	9.74	8.48	delayed	Aspiration
2	Hayashi [12]	MM2C-type sCJD	52	F	58	8	oral	N.D.	N.D.	N.D.	N.D.	N.D.	N.D.	N.D.	N.D.	N.D.
					68	3	oral	N.D.	N.D.	N.D.	poor	N.D.	N.D.	N.D.	preserved	non
					N.D.	4	oral, gastrostomy	assistance	N.D.	N.D.	N.D.	N.D.	N.D.	N.D.	N.D.	N.D.
3	Kunieda [13]	gCJD v1801	69	F	12 M	8	N.D.	N.D.	N.D.	N.D.	N.D.	N.D.	N.D.	N.D.	N.D.	N.D.
					27 M	N.D.	oral	N.D.	sitting	Jelly, liquid, chopped food	slightly poor	N.D.	N.D.	N.D.	not delay	N.D.
					31 M–39 M	N.D.		N.D.	〃	N.D.	slightly worse	N.D.	N.D.	N.D.	delayed	N.D.
					42 M	5	oral, gastrostomy	assistance	N.D.	easy to swallow food	N.D.	N.D.	N.D.	N.D.	N.D.	N.D.
					79 M	N.D.	oral, gastrostomy	assistance	reclining	N.D.	poor	N.D.	N.D.	N.D.	more delayed	non

FILS level 8: the patient eats three meals a day with the exclusion of food that is particularly difficult to swallow; FILS level 7: easy-to-swallow food is orally ingested for three meals. No alternative nutrition is given; FILS level 5: easy-to-swallow food is orally ingested for one to two meals; however, alternative nutrition is also provided; FILS level 4: easy-to-swallow food less than the meal quantity (enjoyment level) is orally ingested; FILS level 3: swallowing training using a small quantity of food is performed.
